# Rebalancing the Neurovascular Axis: NTRK2 Isoform Equilibrium as a Determinant of Pulmonary Endothelial Regeneration

**DOI:** 10.1111/cpr.70200

**Published:** 2026-03-19

**Authors:** Gan Zhou, Cheng Tan, Yifei Miao

**Affiliations:** ^1^ Human Organ Physiopathology Emulation System (HOPE) Institute of Zoology, Chinese Academy of Sciences Beijing China; ^2^ Department of Gynecology Peking University People's Hospital Beijing China; ^3^ Beijing Institute for Stem Cell and Regenerative Medicine Beijing China

## Abstract

Summary of the functions of the NTRK2 receptor during lung injury and regeneration. Our recent work using both human organoid and mouse disease models highlights the non‐conventional role of NTRK2 isoforms in regulating pulmonary vasculature and alveolar regeneration during bronchopulmonary dysplasia.

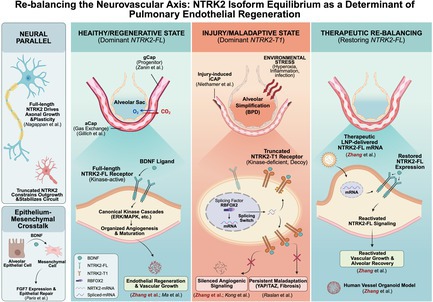

To the Editor,

1

Over the past decade, the lung has emerged as a model for understanding how specialized vascular systems regenerate after injury. Once viewed as a passive gas‐exchange organ supported by epithelial renewal, the lung is now recognized as a tissue where the endothelium itself acts as both a structural scaffold and a regenerative driver. Endothelial cells (ECs) are increasingly recognized as coordinating alveolar growth, inflammatory resolution, and matrix remodelling through lineage‐specific programs. Among the molecular regulators identified, the neurotrophic receptor tyrosine kinase 2 (NTRK2)—known classically as TrkB—has recently redefined how the endothelium senses, interprets, and responds to injury.

Our recent study [1] provides evidence that the regenerative success of pulmonary capillary ECs depends on the balance between NTRK2 isoforms rather than total receptor abundance. Through multiomic and spatial transcriptomic profiling of human bronchopulmonary dysplasia (BPD) lungs and murine hyperoxia models, we discovered that the splicing factor RBFOX2 mediates a switch between the full‐length, kinase‐active receptor (NTRK2‐FL) and the truncated, kinase‐deficient variant (NTRK2‐T1). This isoform shift impairs endothelial regeneration by silencing angiogenic signaling and promoting maladaptive proliferation. Remarkably, restoring NTRK2‐FL expression through lipid nanoparticle (LNP)‐delivered mRNA therapy reactivated vascular growth in iPSC‐derived vessel organoids and reversed alveolar simplification in vivo. These findings identify NTRK2 isoform imbalance as a key driver of endothelial dysfunction and establish isoform‐targeted RNA therapy as a mechanistically specific avenue for vascular regeneration (Figure 1).

**FIGURE 1:**
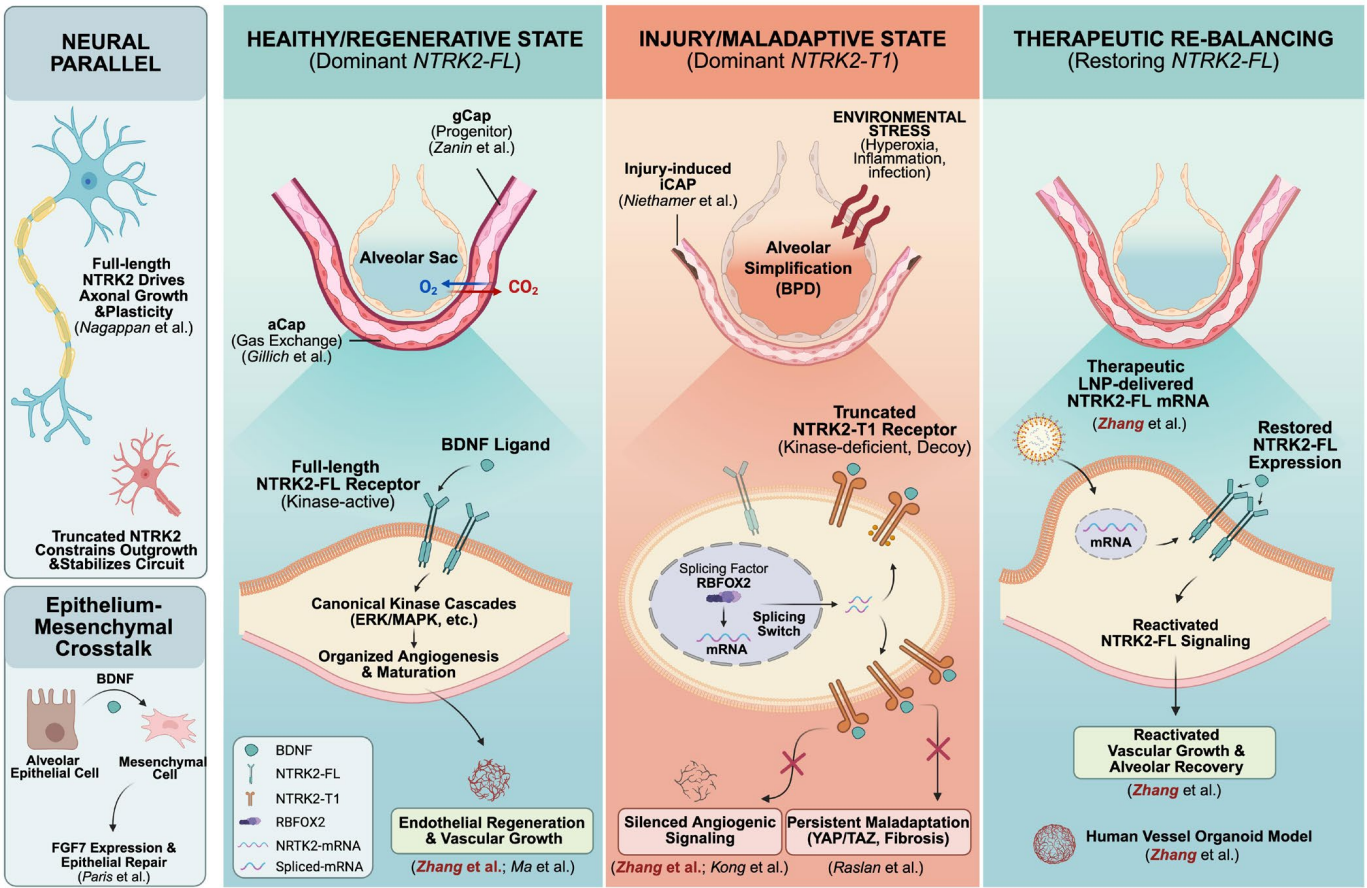
In healthy lungs, the endothelium mainly has the full‐length NTRK2 receptor (NTRK2‐FL). Like epithelial‐mesenchymal crosstalk, where epithelial STAT3 activation induces BDNF secretion, the binding to NTRK2‐FL triggers kinase cascades that promote endothelial regeneration, angiogenesis, and capillary maturation, especially progenitor‐like gCap and aCap cells. Environmental stressors, such as hyperoxia, inflammation, and infection, induce a splicing switch regulated by RBFOX2, shifting toward the truncated, kinase‐deficient NTRK2‐T1 isoform. NTRK2‐T1 acts as a decoy, trapping BDNF and blocking downstream signalling, thereby reducing angiogenesis, alveolar simplification, and persistent maladaptive endothelial states such as iCAPs linked to YAP/TAZ activation and fibrosis. Our recent work with LNP‐delivered NTRK2‐FL mRNA bypasses this splice defect, restoring NTRK2‐FL expression and neurotrophic signalling. This promotes vascular and alveolar recovery, validated in models and human organoids. This mechanism mirrors neurodevelopment, in which full‐length NTRK2 promotes axonal growth, whereas truncated forms stabilize circuits.

2

This discovery is the culmination of a series of convergent advances in pulmonary vascular biology. The first came from developmental single‐cell atlases that defined the molecular heterogeneity of the pulmonary capillary endothelium. Zanini et al. [2] revealed that postnatal alveolarization is orchestrated by two transcriptionally distinct capillary EC lineages: general capillary (gCap or CAP1) cells, marked by *Peg3* and *Plvap* [3], and aerocyte (aCap or CAP2) cells, characterized by *Car4* and *Fibin*. gCap cells exhibit proliferative and progenitor‐like behaviour, serving as the primary source of endothelial renewal, while aCap cells form the specialized gas‐exchange interface [4]. This functional dichotomy—one population proliferative, the other differentiated—establishes the fundamental blueprint of the pulmonary capillary network. Intriguingly, Zanini et al. observed that hyperoxia suppresses angiogenic programs in gCap cells and perturbs aCap maturation, suggesting that developmental stress locks the endothelium in a pre‐reparative but ineffective state [2]. These developmental insights provided the first hint that endothelial subtype composition, rather than total EC abundance, determines regenerative potential.

3

The second conceptual advance arose from recognition that neurotrophic signalling operates far beyond the nervous system. Paris et al. [5] demonstrated that alveolar epithelial STAT3 activation induces secretion of brain‐derived neurotrophic factor (BDNF), which activates Ntrk2 on neighbouring mesenchymal cells, promoting FGF7 expression and epithelial repair. As indicated in various human lung single‐cell transcriptomic atlases scrutinized by LungMAP.net [6, 7, 8], the expression level of BDNF in lung PNEC and neurons is relatively low or undetectable (Figure 2). Taken together, we believe the primary contributor of BDNF is the alveolar epithelial. This unexpected epithelial‐mesenchymal communication established the BDNF‐Ntrk2 axis as a generalized regenerative signalling pathway. Although that study focused on the epithelium, it introduced the idea that the lung parenchyma uses ‘neurotrophic logic’ to coordinate multicellular regeneration.

**FIGURE 2:**
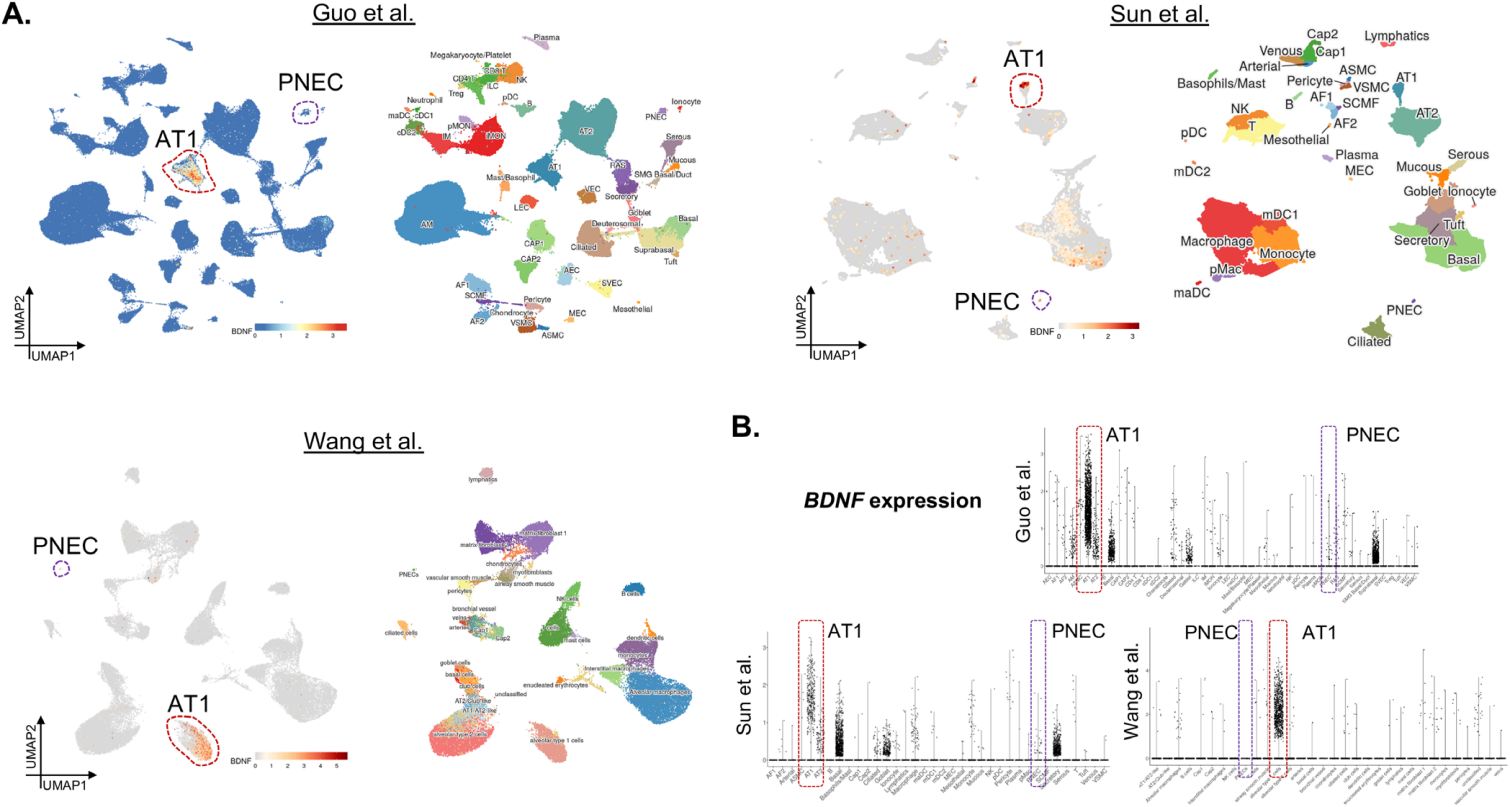
Expression of *BDNF* gene within various human lung cell types. (A) Feature plots of the *BDNF* gene across various human lung single‐cell transcriptomic atlases scrutinized by LungMAP.net. (B) Violin plots showing the *BDNF* gene expression from A.

With the advent of single‐cell transcriptomics, attention turned to the endothelium. Niethamer et al. [9] identified an injury‐induced capillary endothelial population (iCAPs) expressing *Ntrk2* that persisted for months after influenza infection, suggesting that Ntrk2 marks a long‐lived regenerative phenotype. Similarly, Raslan et al. [10] observed that Ntrk2^+^ endothelial cells co‐activate YAP/TAZ and glycolytic programs during injury repair and that this activation fails to resolve in aged or fibrotic lungs. These studies positioned NTRK2 as a marker of endothelial plasticity, linking its expression to both regeneration and maladaptation depending on cellular context.

Yet, the precise role of NTRK2 signalling in EC repair remained paradoxical. Ma et al. [11] found that pharmacologic activation of NTRK2 during neonatal hyperoxia enhanced capillary proliferation and alveolar recovery through ERK/MAPK signalling. Conversely, Kong et al. [12] reported that injury induces a truncated NTRK2‐T1 isoform that lacks catalytic function, marking a proliferative but transient endothelial subset with limited reparative capacity. Niethamer et al. [9] also found that the iCAPs expressed only the kinase‐deficient NTRK2 isoform in influenza‐induced injury. NTRK2 expression was low in healthy human lung tissue but was significantly increased in samples from chronic obstructive pulmonary disease (COPD) and alpha‐1 antitrypsin (AAT) deficiency. The apparent contradiction—that NTRK2 could be both pro‐regenerative and non‐functional—was resolved by our discovery that the relative proportion of NTRK2 isoforms dictates signalling outcomes. When NTRK2‐FL predominates, BDNF engagement activates canonical kinase cascades, promoting angiogenesis and vascular maturation. When NTRK2‐T1 dominates, BDNF becomes a 'silent ligand’, trapped by receptor decoys that block downstream signalling.

This mechanistic insight reframes endothelial plasticity as a problem of isoform equilibrium. The pulmonary endothelium exists in a state of dynamic balance between two transcriptional attractors: a regenerative, kinase‐active configuration defined by NTRK2‐FL and a maladaptive, kinase‐silent configuration defined by NTRK2‐T1. Environmental stress—such as hyperoxia, inflammation, or mechanical ventilation—skews this equilibrium toward the truncated form, limiting recovery. Conversely, restoring NTRK2‐FL re‐establishes the angiogenic trajectory, enabling organized regrowth of gCap networks and restoration of the gCap:aCap ratio. These findings suggest that endothelial fate decisions after injury are encoded not by new transcriptional programs but by reversible splicing dynamics within existing gene networks.

In the broader context of vascular biology, the parallels between the neural and endothelial systems are striking. In neurons, full‐length NTRK2 drives axonal growth and synaptic plasticity, whereas truncated NTRK2 isoforms (particularly NTRK2‐T1) constrain outgrowth and stabilize the circuit [13]. The lung endothelium appears to use the same strategy, with the NTRK2 isoform ratio functioning as a molecular rheostat for vascular remodelling. This shared mechanism underscores a deep evolutionary connection between the nervous and vascular systems—both rely on receptor diversity and post‐transcriptional control to regulate regenerative capacity.

The translational implications are equally compelling. Isoform‐targeted RNA therapy represents a precision approach that restores endogenous regenerative signalling without disrupting lineage identity. In our study, LNP‐mediated delivery of NTRK2‐FL mRNA reinstated angiogenic function in human vessel organoids and reversed capillary rarefaction in hyperoxia‐exposed mice. Beyond BPD, this strategy could extend to adult lung diseases characterized by endothelial exhaustion, such as idiopathic pulmonary fibrosis, pulmonary hypertension, and even long‐term post‐viral syndromes. Furthermore, modulating RBFOX2‐mediated splicing offers an orthogonal pharmacologic route to achieve isoform rebalancing. Small‐molecule inhibitors or antisense oligonucleotides that suppress the NTRK2‐T1 splice variant could, in principle, rejuvenate the endothelium without genetic manipulation.

Importantly, these findings integrate seamlessly with developmental insights [2, 4]. The early postnatal transition from gCap to aCap endothelium involves the gradual silencing of proliferative genes and the upregulation of stability markers, such as *S1pr1* and *Itga1*. Hyperoxia arrests this transition, leaving a pool of immature gCap cells reminiscent of the truncated Ntrk2‐dominant state we describe. Reinstating NTRK2‐FL may thus restore not only angiogenic signalling but also the proper temporal sequence of capillary maturation—essentially re‐enacting the developmental script of vascular growth that injury has interrupted.

Taken together, these converging studies suggest that NTRK2 signalling is one of the master integrators of vascular repair across developmental, acute, and chronic contexts. It links epithelial‐endothelium crosstalk to maintain endothelial homeostasis, couples mechanical and metabolic inputs to molecular signalling, and reconciles regenerative success with failure through isoform dynamics [1, 11, 13]. The activation of endothelial NTRK2 receptor by BDNF, which is secreted prominently by alveolar epithelium, leads to receptor dimerization and autophosphorylation and recruitment of adaptor proteins that engage major signalling pathways, including RAS–MAPK, PI3K–AKT, and PLCγ [14, 15]. These pathways mediate survival, proliferation, and cytoskeletal dynamics for both epithelial and endothelial behaviour. In essence, NTRK2 defines the lung endothelium's capacity to remember and to reset—to respond to injury not merely by proliferating, but by recapitulating the developmental logic of network formation.

Yet, there are currently no registered clinical trials specifically testing NTRK2(TrkB) agonists in BPD patients. However, these findings have paved the road to the treatment of BPD. As suggested, nonspecific NTRK2 agonism may yield transient or context‐dependent benefits but is unlikely to correct the underlying regenerative defect when isoform imbalance predominates. Isoform‐targeted strategies, such as mRNA‐based restoration of NTRK2‐FL or modulation of alternative splicing, therefore represent a more precise and promising approach.

The implications extend beyond the lung. Other organ systems—including the heart, kidney, and brain—contain endothelial subpopulations that express NTRK2 during regeneration. Whether similar isoform‐dependent regulation regulates repair in those tissues remains an open question. If so, the principles uncovered here may represent a universal regenerative axis applicable to diverse vascular beds. The prospect that a single receptor family could underlie both neural plasticity and vascular renewal highlights the profound unity of repair mechanisms across organ systems.

In closing, we propose that the balance between NTRK2‐FL and NTRK2‐T1 serves as a molecular switch implicating endothelial fate. This equilibrium integrates environmental stress, metabolic state, and developmental memory into a single binary decision: regeneration versus remodelling. Understanding and manipulating this balance may yield new therapeutic avenues for vascular repair that rely on restoring endogenous competence rather than delivering exogenous growth factors. The lung, long considered a victim of its delicate capillary architecture, may in fact hold the blueprint for self‐renewal—encoded not in new genes, but in how existing genes are spliced, interpreted, and balanced.

## Author Contributions

Gan Zhou drafted the scientific schematics, and Gan Zhou, Cheng Tan, and Yifei Miao wrote the manuscript.

## Funding

This work was supported by National Key Research and Development Program of China (2025YFA0922000, 2025YFC3408800) and CAS Strategic Priority Research Program (B) (XDB1480200).

## Conflicts of Interest

The authors declare no conflicts of interest.

## Data Availability

Data sharing not applicable to this article as no datasets were generated or analysed during the current study.
